# Improving the Performance of ZnS Photocatalyst in Degrading Organic Pollutants by Constructing Composites with Ag_2_O

**DOI:** 10.3390/nano11061451

**Published:** 2021-05-30

**Authors:** Dequan Yu, Hao Fang, Peikai Qiu, Fancong Meng, Haixia Liu, Shuai Wang, Pingli Lv, Xiaoyan Cong, Qingfen Niu, Tianduo Li

**Affiliations:** 1 School of Chemistry and Chemical Engineering, Shandong Provincial Key Laboratory of Molecular Engineering, Qilu University of Technology, Jinan 250353, China; dequanyu_qlu@hotmail.com (D.Y.); peikaiqiu@hotmail.com (P.Q.); mengfancong2021@hotmail.com (F.M.); qfnchemqlu@hotmail.com (Q.N.); 2 School of Light Industry Science and Engineering, Qilu University of Technology, Jinan 250353, China; qlgdlpl@163.com; 3 Jinan Kuoda Biological Technology CO., LTD, Jinan 250107, China; congxiaoyankuoda@hotmail.com

**Keywords:** water treatment, composite materials, nanomaterials, photocatalysis, organic pollutants

## Abstract

ZnS is a promising photocatalyst in water purification, whereas its low photon efficiency and poor visible-light response restrict its application. Constructing composites may help solve these problems. In this work, Ag_2_O was introduced to ZnS for the first time based on their energy band characteristics to form a novel ZnS/Ag_2_O composite photocatalyst. In the model reaction of degrading methylene blue, the as-designed catalyst exhibited high catalytic activity among a series of ZnS-based composite photocatalysts under similar conditions. The catalytic rate constant was up to 0.138 min^−1^, which is 27.4- and 15.6-times higher than those of ZnS and Ag_2_O. This composite degraded 92.4% methylene blue in 50 min, while the ratios were 31.9% and 68.8% for ZnS and Ag_2_O. Catalytic mechanism study based on photoluminescence and radical-scavenging experiments revealed that the enhanced photocatalytic activity was attributed to the composite structure of ZnS/Ag_2_O. The structure not only facilitated the separation and transmission of photogenerated carriers but also extended the light response range of the catalyst. The as-designed ZnS/Ag_2_O composite is promising in degrading organic pollutants in water.

## 1. Introduction

Zinc sulfide (ZnS) is a promising photocatalyst which has broad applications in environmental fields, especially degrading organic pollutants and water purification [1,2,3,4,5,6,7,8]. As a typical n-type semiconductor, ZnS has received increasing attention, owing to its more negative reduction potential under irradiation [9,10,11,12]. However, improving its catalytic activity is hindered by two challenges: Poor visible-light response for its wide band gap [13] and low photon efficiency for its rapid electron-hole recombination [14]. To solve these problems, many strategies have been explored [15,16,17,18,19]. Among them, combining different types of semiconductors to form composites can improve the transmitting efficiency of photogenerated carriers and extend the spectral response range [20,21,22,23]. Therefore, compositing appropriate semiconductors is an effective approach to enhance the photocatalytic activity of ZnS.

Silver oxide (Ag_2_O), a p-type semiconductor with a narrow band gap, has been widely used as an antibacterial material, colorant, preservative, and electrode material [24,25,26,27]. It has also been a promising photocatalyst since Wang et al. [28,29] discovered its photocatalytic activity. Subsequently, a series of Ag_2_O-involved composites have been reported, such as TiO_2_/Ag_2_O [30], ZnO/Ag_2_O [31], Bi_2_WO_6_/Ag_2_O [32], and g-C_3_N_4_/Ag_2_O [33]. Benefitting from the efficient carrier separation of composites and extended light response range due to the narrow band gap of Ag_2_O, these composites display higher photocatalytic activities than respective components. Therefore, combining n-type semiconductor ZnS with p-type semiconductor Ag_2_O to form a composite may elevate the activity of pristine ZnS.

Herein, Ag_2_O was introduced to ZnS for the first time based on respective energy band characteristics. A novel ZnS/Ag_2_O photocatalyst was constructed and synthesized by a simple chemical precipitation method. The as-designed photocatalyst was composed of n-type semiconductor ZnS and p-type semiconductor Ag_2_O, exhibiting higher catalytic activities than pristine ZnS or Ag_2_O toward degrading the model pollutant methylene blue (MB). Furthermore, the composite exhibited a high-rate constant of 0.138 min^−1^, indicating a high catalytic activity among a series of ZnS-based photocatalysts under similar conditions. Catalysis mechanism study of the enhanced activity revealed that the ZnS/Ag_2_O composite not only facilitated the separation and transmission of photoelectrons and holes but also extended the light response range of the photocatalyst. The as-designed ZnS/Ag_2_O composite is a promising photocatalyst for degrading hazardous organic pollutants in the water purification application.

## 2. Materials and Methods

### 2.1. Chemicals

Zinc nitrate hexahydrate (Zn(NO_3_)_2_·6H_2_O, A.R.), thiourea (CH_4_N_2_S, A.R.), polyvinylpyrrolidone (PVP, A.R.), and sodium hydroxide (NaOH, A.R.) were purchased from Sinopharm Chemical Reagent Co., Ltd. (SCRC, Beijing, China). Silver nitrate (AgNO_3_, A.R.) was supplied by Sigma Aldrich (Merck KGaA, Darmstadt, Germany). All chemicals were used without further purification. Aqueous solutions were prepared using deionized water.

### 2.2. Synthesis of ZnS Broccoli-Like Microspheres

ZnS broccoli-like microspheres were synthesized through the hydrothermal process. In a typical process, a certain amount of Zn(NO_3_)_2_ (0.1 M), CH_4_N_2_S (0.3 M), and 0.05 g PVP were mixed together, and stirred for 30 min. The suspension was transferred into a Teflon-lined autoclave and kept at 120 °C for 12 h. The final products were collected by centrifugation, washed with ethanol, and deionized water for several times. Then, the products were dried in an oven at 60 °C for 6 h. The products obtained under other temperatures were synthesized under corresponding temperatures while keeping other conditions consistent.

### 2.3. Synthesis of ZnS/Ag_2_O Composite

In a typical process, 0.2 g as-prepared ZnS was dispersed in 50 mL deionized water, and 0.117 g AgNO_3_ was added to the suspension. After vigorous stirring for 30 min, a certain amount of 2 M NaOH was added dropwise to the suspension until it reached a pH of 14. Then, the composite was collected by centrifuging at 5000 rpm. The composite was then washed with deionized water and dried at 60 °C. In addition, the pristine Ag_2_O was synthesized through a similar approach at room temperature, which was used as a contrast sample.

### 2.4. Characterizations of Samples

The crystallographic phases of samples were characterized by X-ray diffraction (XRD) using a Bruker D8 Advance X-ray powder diffractometer (Bruker Corp., Billerica, MA, USA) with Cu-Kα radiation (λ = 1.5418 Å). Morphological observations were performed by a Hitachi S-4800 field emission scanning electron microscope (SEM, Tokyo, Japan). The diffuse reflectance spectra (DRS) were recorded by a Shimadzu UV-2600 spectrophotometer equipped with an integrating sphere, using BaSO_4_ as the reflectance standard. Photoluminescence (PL) spectra were obtained by an Edinburgh FLS920 fluorescence spectrometer (Edinburgh Instruments Ltd., Livingston, England) under an excitation wavelength of 325 nm.

### 2.5. Photocatalytic Activity Tests

The photocatalytic activities of the as-prepared samples were evaluated in terms of the model reaction of degrading MB. The 300 W xenon lamp (PLS-SXE300UV, Perfectlight Co., Ltd., Beijing, China) was positioned 10 cm away from the cuvette and was used as light source to trigger the photocatalytic reaction. In a typical test, 0.05 g of a certain sample was dispersed into 100 mL MB solution (10 mg/L) with magnetic stirring. The suspension was stirred constantly in the dark for 1 h to achieve the absorption equilibrium before irradiation. The residual concentration of MB was determined by UV-2600 UV-Vis spectroscopy (Shimadzu, Kyoto, Japan).

## 3. Results and Discussion

**Figure 1:**
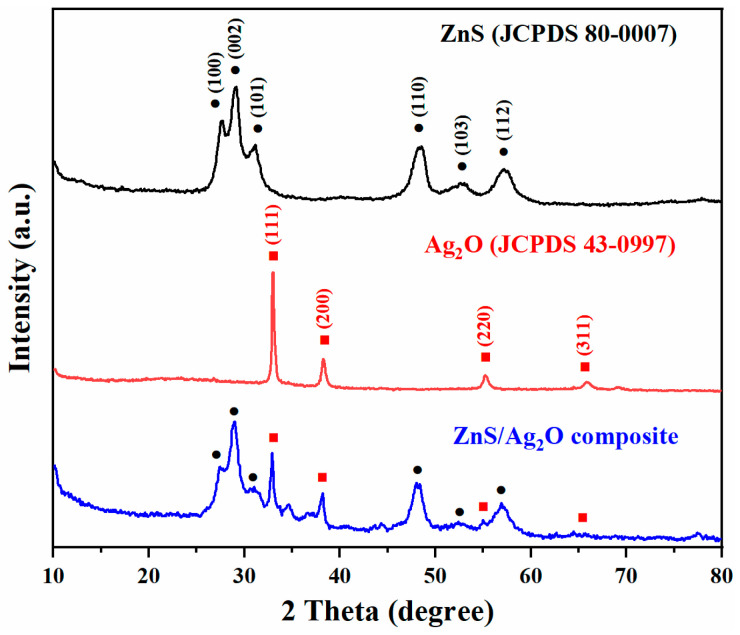
XRD patterns of the as-prepared ZnS, Ag_2_O, and ZnS/Ag_2_O composite. Standard XRD peaks of ZnS (JCPDS 80-0007) and Ag_2_O (JCPDS 43-0997) are marked.

Figure 1 shows the XRD patterns of the as-prepared ZnS, Ag_2_O, and ZnS/Ag_2_O composite. From the XRD pattern in black, it is evident that all the diffraction peaks can be well-indexed to the hexagonal wurtzite structure of ZnS (JCPDS 80-0007), with characteristic diffractions of the (100), (002), (101), (110), (103), and (112) crystal planes. The pattern in red can be assigned to the Ag_2_O in cubic phase (JCPDS 43-0997). The typical peaks in good crystallinity of Ag_2_O can be clearly identified as (111), (200), (220), and (311) facets. For the ZnS/Ag_2_O composite, the XRD pattern is composed of two sets of characteristic peaks, which are the hexagonal ZnS and cubic Ag_2_O phase. In addition, there is no characteristic peak relevant to another phase, which demonstrates the high purity of the product. Therefore, the as-prepared typical sample is indeed a composite composed of ZnS and Ag_2_O.

**Figure 2:**
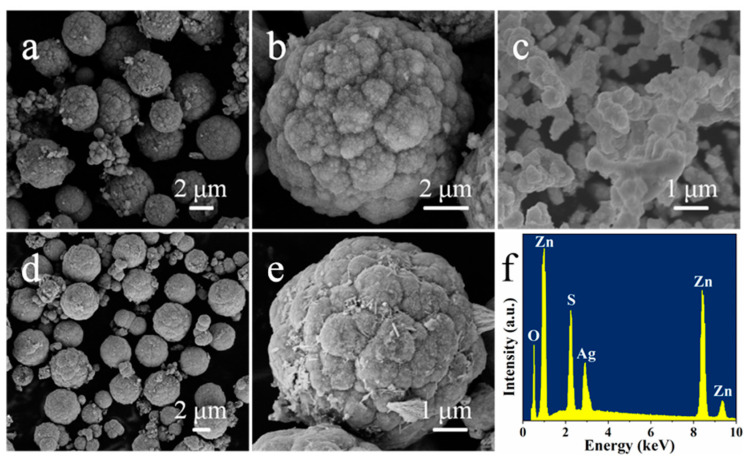
(**a**,**b**) SEM images of ZnS at different magnifications, (**c**) SEM image of Ag_2_O, (**d**,**e**) SEM images of ZnS/Ag_2_O composite at different magnifications, (**f**) EDX spectrum of ZnS/Ag_2_O composite.

The details of morphology and microstructure of as-prepared ZnS, Ag_2_O, and ZnS/Ag_2_O composite were studied by SEM. Figure 2a,b show the typical images of pristine ZnS at different magnifications. As-prepared ZnS are shown as broccoli-like microspheres with an average size around 3–4 μm. Moreover, the microspheres had rough surfaces, providing a plenty of sites for Ag_2_O to nucleate and grow and facilitating the formation of the ZnS/Ag_2_O composite. Figure 2c displays an SEM image of pristine Ag_2_O which has a morphology with irregular aggregation of lots of nanoparticles with a diameter about 200–300 nm. Figure 2d,e exhibit the morphology of ZnS/Ag_2_O composite at different magnifications. The morphology of ZnS was not affected and still maintained the original broccoli-like microspheres after composition with Ag_2_O. The Ag_2_O nanoparticles were uniformly covered on the surface of ZnS microspheres, constituting the ZnS/Ag_2_O composite. In addition, EDX spectrum of ZnS/Ag_2_O composite was acquired simultaneously with SEM, as displayed in Figure 2f. The characteristic X-ray peaks of Zn, Ag, S, and O can be found without other element emerges, revealing the elemental composition of the composite.

**Figure 3:**
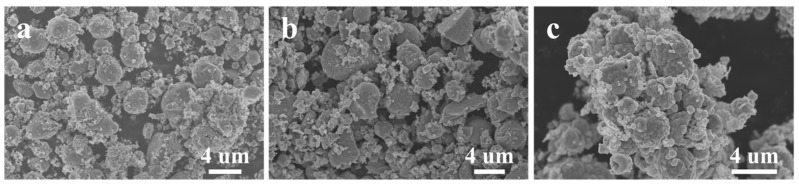
SEM images of ZnS obtained at different temperatures: (**a**) 90 °C, (**b**) 150 °C, (**c**) 200 °C.

To explore the law of morphology evolution, condition control experiments on morphologies were carried out. Among these influencing factors, temperature showed more obvious regularity. Figure 3a displays the ZnS microspheres obtained at a temperature of 90 °C while the other experiment conditions were kept identical to the typical sample (120 °C). The as-obtained product was mainly ZnS microspheres with plenty of nanoparticles. The diameter of the microspheres was around 3 μm. As the temperature increased to 150 °C (Figure 3b), the morphology of the product did not change significantly, remaining spherical. However, the proportion of ZnS microspheres decreased while that of the nanoparticles increased. Besides, the diameter of ZnS microspheres became uneven. In Figure 3c, at a temperature of 200 °C, there were no separate ZnS microspheres in the product. Agglomerates emerged that were composed of ZnS microspheres, and the shape of the ZnS microspheres became blurred and uneven in size.

**Figure 4:**
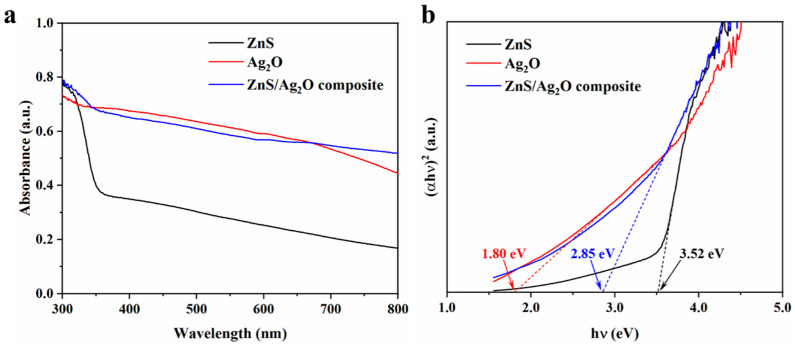
(**a**) UV-Vis DRS spectra and (**b**) Tauc plots of ZnS, Ag_2_O, and ZnS/Ag_2_O composite to show their band gap energy values.

To investigate whether the introduction of Ag_2_O improved the light absorption properties of ZnS, UV-Vis DRS and derived Tauc plots were acquired to obtain the band gap energy for the ZnS, Ag_2_O, and ZnS/Ag_2_O composite. As-obtained plots are shown in Figure 4a. The absorption edge of pristine ZnS was around 340 nm in the UV region, while the pristine Ag_2_O exhibited strong light absorption performance both in the UV and visible light regions, which is ascribed to its much lower energy for band gap transition. Combining the light absorption properties of ZnS and Ag_2_O in UV and visible light regions of 300–800 nm, the ZnS/Ag_2_O composite achieved a great elevation in light absorption in visible light regions, forming an obvious contrast to pristine ZnS. Figure 4b shows the Tauc plot of the three samples. The band gap energy of semiconductors can be obtained from the *x*-axis intercept of the extended line. From the graph, the band gap energy values for the ZnS, Ag_2_O, and ZnS/Ag_2_O composite were determined to be 3.52 eV, 1.80 eV and 2.85 eV, respectively, which suggests that the band gap energy of ZnS was greatly reduced after introducing Ag_2_O to ZnS to form the composite. In particular, the band gap energy changed from 3.52 eV to 2.85 eV. The results demonstrate huge improvement by the introduction of Ag_2_O, which led to a higher photon efficiency and extended the light response range of the composite.

**Figure 5:**
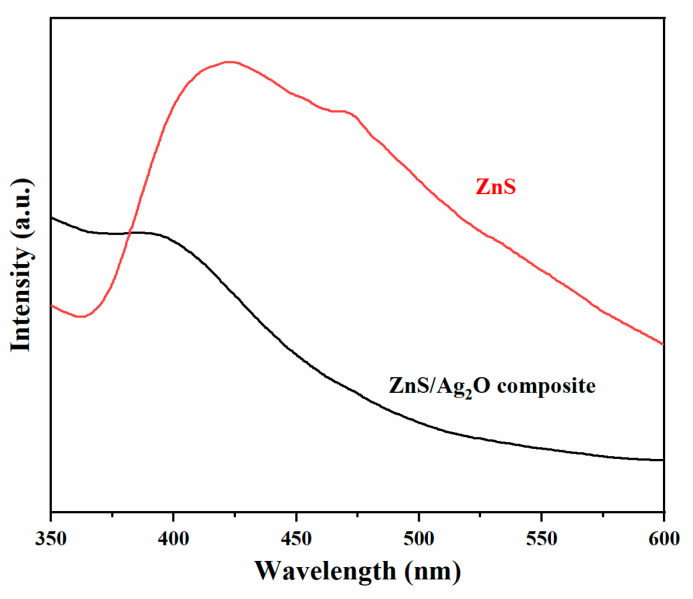
PL spectra of ZnS and ZnS/Ag_2_O composite.

For photocatalysts, PL spectrum is crucial because it is related to the recombination of photogenerated electrons and holes. High fluorescence intensity means that there will be less carriers involved in the photocatalytic reaction, resulting in a low quantum efficiency of catalysts. Under the exciting wavelength of 325 nm, PL spectra of pristine ZnS and ZnS/Ag_2_O composite were obtained, as shown in Figure 5. The ZnS showed two strong emission peaks at 423 nm and 472 nm. For semiconductors, two emission peaks were observed, corresponding to interstitial and trapped surface state emission, respectively. The emission peak at 423 nm is evidence of sulfur vacancies due to the recombination of electrons from shallow trap state to the sulfur vacancies, which is consistent with the results reported previously [34]. The emission peak centered at 472 nm can be attributed to the zinc vacancies in ZnS lattice. After the addition of Ag_2_O, the fluorescence intensities of ZnS/Ag_2_O composite decreased greatly, indicating that photogenerated electrons and holes can rapidly migrate between ZnS and Ag_2_O, thus suppressing the recombination of carriers.

**Figure 6:**
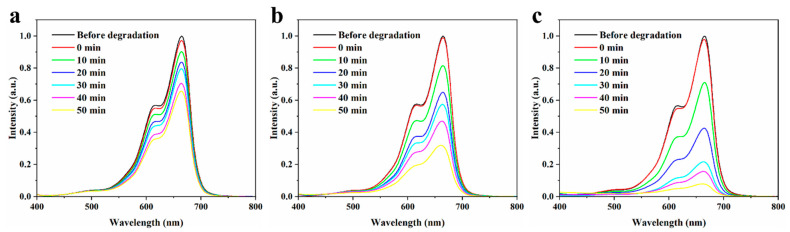
UV-Vis absorption spectra of MB in the presence of (**a**) ZnS, (**b**) Ag_2_O, and (**c**) ZnS/Ag_2_O composite recorded at different time during the photocatalytic tests.

The photocatalytic activity of obtained ZnS/Ag_2_O composite was evaluated by the model reaction of degrading MB under UV-Vis irradiation. For comparison, tests of ZnS and Ag_2_O were also conducted under the same condition. Before irradiation, the solution of MB was stirred evenly with certain catalyst in the dark for 60 min to achieve the absorption-desorption equilibrium. The original UV-Vis spectra data of the three typical samples recorded at different time during the tests are plotted in Figure 6. The data exhibit the overlapped UV-Vis absorption spectra with a major peak at 664 nm, which indexed to the characteristic absorption peak of MB. In Figure 6a, the peak of MB decreased slowly and was catalyzed by ZnS, and the MB was degraded 31.9% in 50 min. However, as shown in Figure 6b, the Ag_2_O catalyst showed a faster degradation rate than ZnS. The peak at 664 nm decreased by 68.8% within the same time, whereas the ZnS/Ag_2_O composite exhibited the highest activity. The concentration of MB decreased rapidly, and 92.4% of MB was degraded in 50 min. The results demonstrate that the ZnS/Ag_2_O composite had a better performance than both of its components. The superior performance of the photocatalyst can be attributed to the improved visual light response of the composite and its higher photon efficiency.

**Figure 7:**
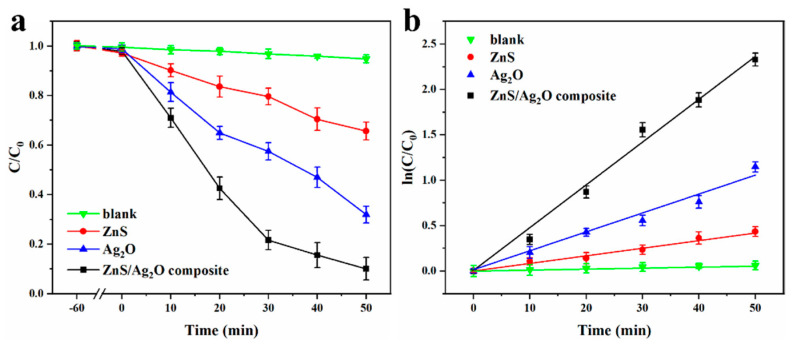
(**a**) Photocatalytic degradation profiles of MB at 664 nm as a function of time. (**b**) Linear fittings of degrading kinetics under different catalytic conditions.

Figure 7 shows the degradation profiles of MB at 664 nm as a function of time. To quantitatively compare the catalytic efficiencies of the above samples, kinetic calculation was carried out. In terms of the Langmuir–Hinshelwood model, degradation can be regarded as a first-order reaction at low MB concentration. Hence, the fitting calculation of degrading kinetics can be simplified to be linear. Besides, this reaction satisfies the apparent first-order reaction rate equation: *ln*(C/C_0_) = −*k*·t, where *k* is the apparent rate constant of first-order reaction, and *ln*(C/C_0_) is a function of irradiation time t. Based on this simplified model, relevant fitting results were obtained, as summarized in Figure 7. The calculated *k* values of ZnS and Ag_2_O are 0.049 min^−1^ and 0.024 min^−1^, respectively, while that of ZnS/Ag_2_O composite is 0.138 min^−1^, which is 27.4- and 15.6-times higher than those of ZnS and Ag_2_O. The reaction cannot proceed without a catalyst under UV-Vis irradiation. The results demonstrate that the as-produced ZnS/Ag_2_O composite has a greatly enhanced photocatalytic activity compared to pristine ZnS and Ag_2_O.

To further evaluate the composite photocatalyst we designed, some relevant reports were retrieved to assess similar catalytic systems for horizontal comparison. All of the selected catalysts used composite photocatalysts with ZnS as the main body, and the catalytic degradation of MB was used as the model reaction to investigate the ability to purify organic pollutants in water. Table 1 lists the catalytic activities of some representative ZnS-based photocatalysts with crucial catalytic properties. The rate constant of the catalyst reported in this work is also listed at the bottom of the table to facilitate horizontal comparison. It can be clearly seen that the as-prepared ZnS/Ag_2_O composite has a high-rate constant among a various of photocatalysts, indicating that it has higher photocatalytic activity than most existing photocatalysts. Therefore, it can be concluded that as-designed ZnS/Ag_2_O composite is a promising candidate in the field of wastewater purification.

**Figure 8:**
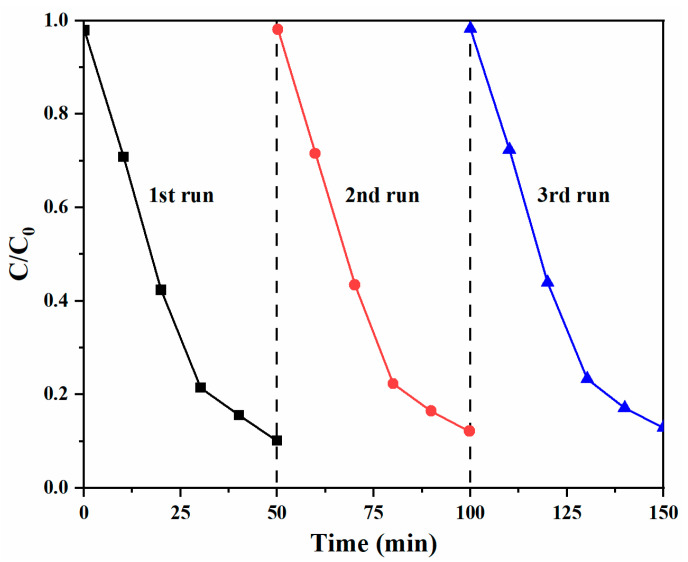
Recycling performance of the ZnS/Ag_2_O composite for degrading MB.

In addition, the recyclability of ZnS/Ag_2_O composite in MB degradation reaction under UV-Vis irradiation was studied, as shown in Figure 8. After three successive cycles, the sample retained nearly consistent photocatalytic efficiency without apparent deactivation, indicating that the ZnS/Ag_2_O composite is stable during the degrading process.

**Figure 9:**
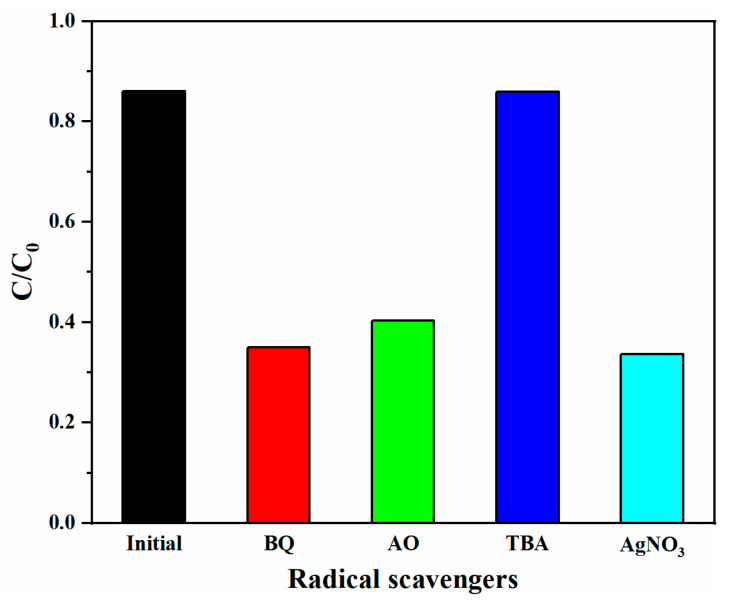
Photocatalytic degradation of MB by the ZnS/Ag_2_O composite alone and with different radical scavengers.

To reveal the photocatalytic mechanism, the main active species in the MB degradation reaction were investigated. Radical scavengers, such as benzoquinone (BQ), ammonium oxalate (AO), tertiary butanol (TBA), and AgNO_3_, have been used to scavenge superoxide radical anions (•O^2−^), holes (h^+^), electrons (e^−^), or hydroxyl free radicals (•OH) respective [35,36]. From Figure 9, the addition of TBA had no obvious influence on the degradation of MB, implying that •OH are not the primary active species. When BQ and AgNO_3_ were added, the reaction rates were dramatically decelerated, indicating that •O^2−^ has a crucial role in the photocatalytic process (O_2_ + e^−^ → •O_2_^-^). Besides, the addition of AO also remarkably suppressed the reaction, suggesting that h^+^ is involved in the degradation process.

**Figure 10:**
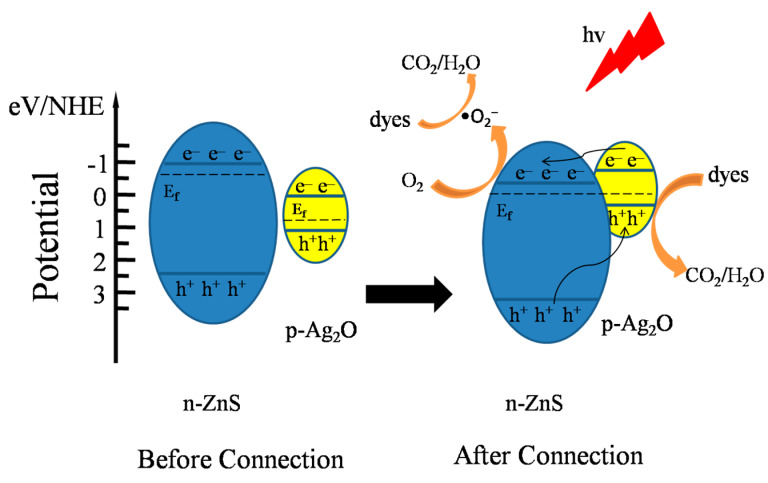
Mechanism for the enhanced photocatalytic activity of the ZnS/Ag_2_O composite.

Based on the above results, a possible mechanism for the enhanced photocatalytic activity of ZnS/Ag_2_O composite was proposed (Figure 10). The activity of a semiconductor photocatalyst mainly depends on the oxidation-reduction potentials of the valence band (VB) and conduction band (CB). Fermi level (E_f_, dashed line in Figure 10) is the chemical potential of thermodynamic equilibrium. Before n-type semiconductor ZnS and p-type semiconductor Ag_2_O formed the composite, their Fermi levels had different potentials. When the ZnS/Ag_2_O composite is formed and irradiated by UV-Vis light, the photogenerated electrons are transferred from ZnS to Ag_2_O due to the initially higher CB potential of ZnS until the quasi-Fermi level (quasi-static equilibrium) is generated [37,38,39,40]. This causes the energy bands of ZnS to shift downward and those of Ag_2_O to shift upward, eventually making the Fermi levels of the two components equal. Because the Fermi level of ZnS is close to CB but that of Ag_2_O is close to VB [39], the final CB position of Ag_2_O is higher than that of ZnS [41]. Therefore, the photogenerated electrons transfer from Ag_2_O to ZnS, driven by the potential difference. Then, the photogenerated electrons reduce the surface chemisorbed O_2_ to form oxidizing species •O_2_^−^, which can degrade MB into small molecules [41,42]. Conversely, photogenerated holes on the VB of ZnS can be transferred to Ag_2_O, which can be consumed to oxidize MB directly. In addition, pristine ZnS has a poor ability for visible-light response due to its high band gap energy. Ag_2_O, having a much lower band gap energy, can effectively extend the spectral response range to visual light, elevate the photon efficiency, and improve the activity by forming a composite with ZnS. Therefore, the photocatalytic performance can be greatly enhanced because photogenerated electrons and holes are more efficiently generated and transferred by the ZnS/Ag_2_O composite.

## 4. Conclusions

In summary, a novel photocatalyst composed of n-type semiconductor ZnS and p-type semiconductor Ag_2_O was designed and constructed by a simple chemical precipitation method. The as-obtained ZnS/Ag_2_O composite exhibited a high photocatalytic activity and favorable stability among a series of similar ZnS-based composite photocatalysts toward degrading MB. The catalytic rate constant reached up to 0.138 min^−1^, which is much higher than pristine ZnS or Ag_2_O. Results of catalytic mechanism experiment revealed that the enhanced catalytic activity can be attributed to the efficient separation and transmission of photogenerated carriers and extended light response range brought about by the narrow band gap of Ag_2_O. The as-designed ZnS/Ag_2_O composite is a promising photocatalyst for removing hazardous organics from wastewater due to its high performance.

## Figures and Tables

**Table 1:** A series of representative ZnS-based photocatalysts in various structures and corresponding catalytic rate constants in the reaction of degrading MB.

No.	Catalysts	Structures	Rate Constants ^1^	Ref.
1	ZnS/CdS	core/shell structure	0.119 min^−1^	[2]
2	ZnS/TiO_2_	nanospheres	0.110 min^−1^	[3]
3	ZnS/ZnO	porous nano-crystal films	0.041 min^−1^	[4]
4	Ag@AgI/ZnS	microspheres	0.031 min^−1^	[5]
5	ZnS/SnO_2_	nanospheres	0.046 min^−1^	[6]
6	ZnS/CuS	supported on reduced graphene oxide	0.159 min^−^^1^	[7]
7	ZnS/MoS_2_	layered structures	0.183 min^−1^	[8]
8	ZnS-Fe_2_O_3_	supported on reduced graphene oxide	0.077 min^−1^	[9]
9	ZnS-ZnIn_2_S_4_	porous hierarchical spherical structure	0.041 min^−1^	[10]
10	ZnS/Ag_2_O	broccoli-like microspheres with nanorods	0.138 min^−1^	this work

^1^ The data of rate constants in s^−1^ were all converted into min^−1^ for convenience to compare.

## Data Availability

Data supporting this study are available within the article.
